# The Supportive Care Needs of Cancer Patients: a Systematic Review

**DOI:** 10.1007/s13187-020-01941-9

**Published:** 2021-01-25

**Authors:** Madeleine Evans Webb, Elizabeth Murray, Zane William Younger, Henry Goodfellow, Jamie Ross

**Affiliations:** 1 UCL Research Department of Epidemiology & Public Health, 1-19 Torrington Place, London, WC1E 6BT UK; 2 Department of Primary Care and Population Health, Upper 3rd Floor, Royal Free Hospital, Rowland Hill Street, London, NW3 2PF UK

**Keywords:** Supportive care, Cancer, Patient needs, Holistic

## Abstract

Cancer, and the complex nature of treatment, has a profound impact on lives of patients and their families. Subsequently, cancer patients have a wide range of needs. This study aims to identify and synthesise cancer patients’ views about areas where they need support throughout their care. A systematic  search of the literature from PsycInfo, Embase and Medline databases was conducted, and a narrative. Synthesis of results was carried out using the Corbin & Strauss “3 lines of work” framework. For each line of work, a group of key common needs were identified. For illness-work, the key needs idenitified were; understanding their illness and treatment options, knowing what to expect, communication with healthcare professionals, and staying well. In regards to everyday work, patients wanted to maintain a sense of normalcy and look after their loved ones. For biographical work, patients commonly struggled with the emotion impact of illness and a lack of control over their lives. Spiritual, sexual and financial problems were less universal. For some types of support, demographic factors influenced the level of need reported. While all patients are unique, there are a clear set of issues that are common to a majority of cancer journeys. To improve care, these needs should be prioritised by healthcare practitioners.

**Supplementary Information:**

The online version contains supplementary material available at 10.1007/s13187-020-01941-9.

## Introduction

### Rationale

Over 42 million people worldwide are currently living with cancer [1]. A cancer diagnosis often results in biographical disruption [2] and distress [3], sometimes lasting years post-treatment [4, 5]. As survival rates continue to increase, more individuals will have to live with the long-term implications of cancer. It is therefore important that the support offered to cancer patients improves to meet this growing demand.

Cancer care pathways are often spread across multiple facilities and delivered by healthcare practitioners (HCP), which make it challenging for a patient’s wider support needs to be met. This has an impact on patient wellbeing [6] and survival outcomes [7, 8]. Many studies focus on the needs of specific patient groups, defined by diagnosis, treatment or demographics, but there is no broad consensus on how common or dissimilar patients’ supportive care needs are across types of cancer and populations. The aim of this study was to synthesise existing data on the support needs of cancer patients across populations. Identifying the common underlying needs of cancer patients, as well as needs that are specific to a patient’s diagnosis or background, will help HCPs provide comprehensive support more efficiently.

## Methods

### Eligibility Criteria

#### Population

Inclusion criteria: Any patient undergoing treatment for any form of cancer. Patients in remission or recovery were eligible only if they had not been in remission for longer than 5 years, a key milestone in cancer survivorship [9].

### Intervention and Comparator

Patients that had received any form of treatment, be it curative or palliative, could be included. As this review was not assessing the effectiveness of an intervention program, there was no appropriate comparator or control group.

### Outcomes

The primary outcome was the identification of any supportive care needs, categorised into emotional, informational, spiritual, social or “other”. Needs could be specifically identified, or could be inferred from reported distress, e.g. patients reporting high levels of loneliness would be categorised as having an emotional need.

### Study Type

Inclusion criteria: Any study design which included collection of primary data, quantitative or qualitative, was eligible for inclusion.

Exclusion criteria: Papers which did not include new primary data (e.g. reviews, meta-analyses, editorials), had not been peer reviewed or were not available in English.

### Search

The search strategy was the keywords: [emotional need] or [spiritual need] or [social need] or [emotional need] AND [Neoplasm(s)] either appearing in the title, abstract, subject heading, keyword heading, protocol supplementary concept, rare disease supplementary concept or as a unique identifier.

The search was carried out on PsycInfo, Embase and Medline databases, on 24 April 2018. This selection was based on a review of which databases have the highest recall rate, while also needing to produce a manageable number of results [10].

Reference lists of included papers were searched for potentially eligible studies.

### Study Selection

Titles and abstracts were screened against the inclusion/exclusion criteria, and 10% of papers were also screened by a second author. For any paper that could not be confidently excluded, the full paper was read to determine whether it should be included. There was 100% agreement between the screeners about which papers should be excluded.

### Data Extraction and Management

Data were extracted into an extraction form, which was piloted and refined. Data extracted from each paper were as follows: title, year of publication, country of study setting, study design, population studied, methods of data collection and analysis and results. The needs identified in each paper were classified as informational, emotional, spiritual, social or other. For quantitative data, scores or rankings for each need were recorded, along with whether needs differed between sub-groups. For qualitative data, overarching themes, subthemes and illustrative quotes were extracted.

### Data Synthesis

Data were analysed using a narrative synthesis method [11]; this allowed for the synthesis of qualitative and quantitative data and analysis of whether medical or demographic factors shaped patient needs [11, 12].

The first step was to group the needs identified in the papers into the categories specified in the primary literature. Seven categories of need were identified in the included papers: emotional, sexual, spiritual, social, financial, daily living, nutritional and informational. The second step was to map these categories onto the Corbin & Strauss “Three lines of work” model of chronic disease management. The model identifies three types of work associated with managing a long-term condition: illness-related work, everyday life work and biographical work [13]. Within each group, the relative importance and prevalence of all the needs identified in the primary literature were compared to identify which were the most common and urgent.

Our goal was to clarify the commonality of the experience of “cancer”, irrespective of the type of cancer, thus providing an overview of the common and important support needs faced by people with cancer, and hence an understanding of where supportive care is most needed. In instances where there was conflicting evidence in the primary literature on the importance of a specific need, clinical and demographic differences between study populations were reviewed in order to understand the potential reasons for this conflict.

The Corbin & Strauss model was chosen because the categories of need identified in the primary literature clearly corresponded to the types of work in the model (Fig. 2). Using the model as a framework to synthesise the data allowed us to compare the relative importance of needs from different categories that fell under the same type of work. The simplicity of the model meant it could be consistently applied to needs that were identified and categorised using a number of different methodologies.

## Results

### Study Selection

**Fig. 1:**
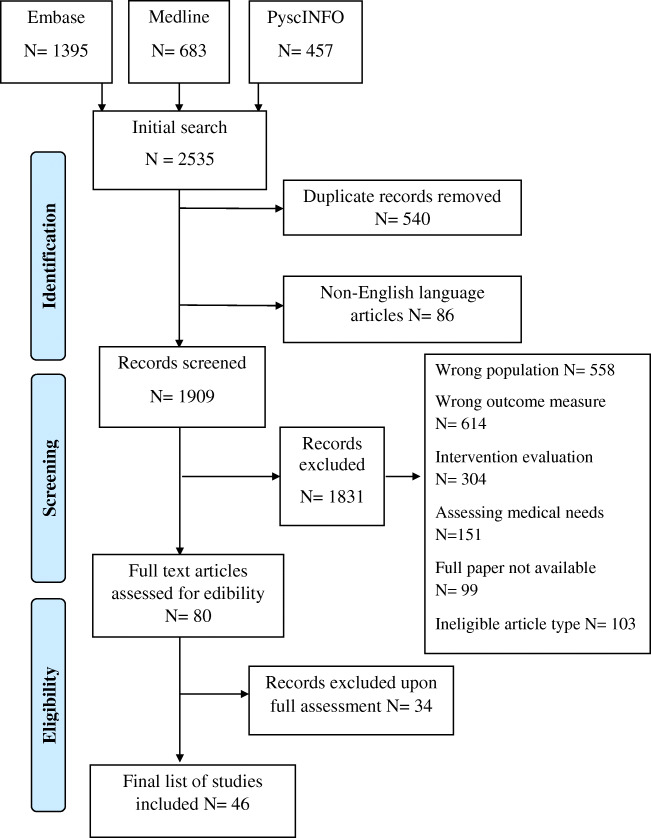
PRISMA flow diagram of the paper identification process

In total, 2535 papers were identified, and 540 duplicates were removed. After screening against the criteria, 1829 papers were removed, and the remaining 80 papers were read in full (Fig. 1). Forty-six papers were found to be eligible for inclusion in this review.

### Study Characteristics

Of the 46 studies, 34 were quantitative, 10 were qualitative and two were mixed methods. Study population sizes ranged from 7 to 1059 participants. Fifteen papers focused on patients with a specific type of cancer, with breast and colorectal cancer being the most common. Three studies looked at patients from specific ethnic backgrounds. Eight papers focused on patients receiving a specific form of care/treatment. Three papers focused on children or young adults. Three papers looked at adults within specific age groups. Eleven studies only included patients at a certain stage of cancer or time since diagnosis. Thirty-nine studies took place in high-income countries, 6 were from middle income countries and 1 took place in a low-income country.

### Needs of Cancer Patients

Thirty-two papers mentioned informational needs, 31 mentioned emotional needs, 24 mentioned spiritual needs and 19 mentioned social needs. Thirty-five papers mentioned needs in at least one of these other categories: nutritional, sexual, daily living or financial.

**Fig. 2:**
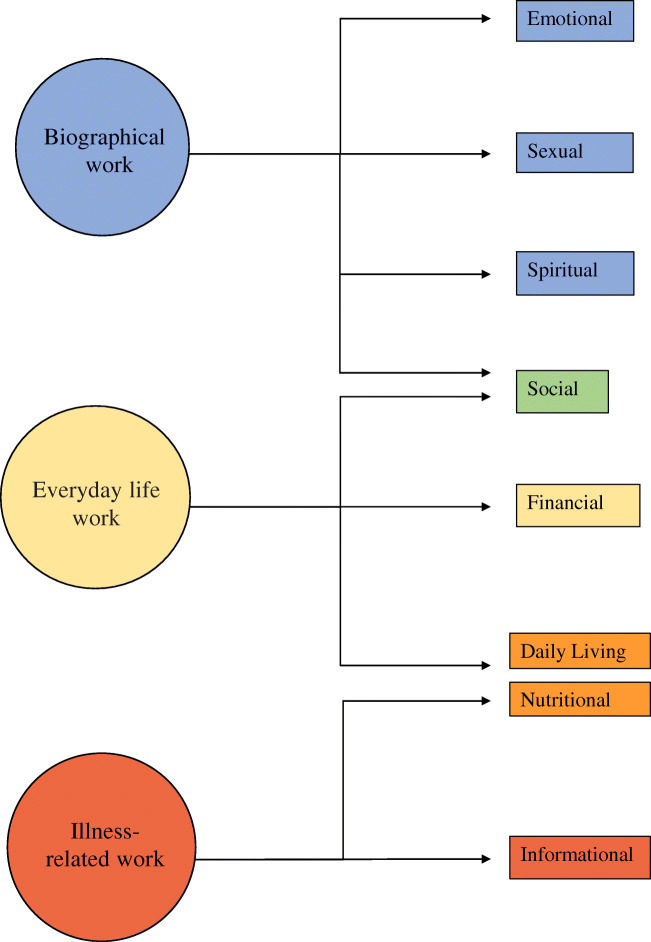
Illustration of how the different domains of need identified fit into Corbin and Strauss’ 3 lines of work model of managing chronic illness

The resulting needs identified were grouped according to the different forms of chronic disease “work” defined by the Corbin & Strauss framework (Fig. 2).

### Illness-Related Work

Illness-related work, defined by Corbin & Strauss, is “the tasks of controlling symptoms; monitoring, preventing crises; carrying out regimens and managing limitations of activity” [13]. The central goal of illness-related work for patients is to understand their illness and treatment, and subsequently the need for information is consistently reported as a high priority [14–29]. The only paper that did not find a high level of informational need specifically measured *unmet* need [30].

Most frequently patients wanted to know what treatment they were receiving and how it worked [20, 26, 27, 29, 31–36], why that treatment had been selected, its effectiveness and its pros and cons [14, 20–24, 26, 35]. Patients also frequently searched for more specific information about their diagnosis and prognosis [15, 20–25, 29, 31, 33, 34].

Patients wanted to know what to expect from their illness and treatment [15, 16, 31, 33–35, 37, 38] (Box 1). This included knowing about the chance of a relapse [26], the length of their hospital stay [32] and when their life would return to “normal” [26, 31, 33, 39]. One paper reported that being given “vague” answers by HCPs frustrated patients [38].

**Table:** 

**1- Knowing what to expect** Beaver, 2010. Colorectal cancer patients - *“I’ve learned myself really… trial and error.”* *“I don’t think there was enough information about what sort of nutrition you need… I have made a point of finding out about this through reading magazines and through the internet”* Beaver, 2016. Breast cancer patients- *“Nobody prepared me for that at all, that aspect of it.”*	

In regard to treatment, patients most often wanted to know what the possible side effects were [16, 18, 21, 22, 25, 27, 31, 33, 37, 38, 40, 41] and how they could manage or relieve them [14, 17, 19, 22–24, 26, 31, 33, 35, 42, 43]. The importance of this information may depend on the stage of the patient’s treatment, as patients receiving follow-up or palliative care placed less importance on symptom management [25, 27].

Wanting to minimise the impact of side effects speaks to a commonly reported desire among patients to be as healthy as possible [14, 15, 18, 19, 22–24, 26–29, 33, 38–40]. This aim is also seen in the nutritional needs of patients [16, 20, 33, 40, 41, 44]. Rather than receiving generic information about healthy diets, patients wanted more specific advice around foods that could aid recovery or minimise side effects [16, 40, 41]. Nutritional needs had an outsized importance in studies involving Native American patients and colorectal cancer patients [16, 40]. For colorectal cancer patients, nutritional needs are likely higher as their cancer directly affects their digestive system. Within the Native American population, there was a strong interest in information about traditional foods, possibly due to culturally specific reasons [40].

Generally, patients wanted their test results as soon as possible [21, 22, 24, 27, 33, 43] and wanted the meaning of the results explained to them [21, 22, 26, 34, 43]. The importance of this information to patients could be due to a desire to have some say in the treatment they are given [18, 33, 34, 45], although the level of interest in alternative treatments varied significantly [14, 18, 24, 40, 44] (Box 2). The only study where information about tests was less important involved newly diagnosed patients [18].

**Table:** 

**2 - Being involved with treatment decisions** Tamburini, 2003.- *“speaking about it together with him/her could help to find a more adaptive solution to my demands, perhaps changing a little of what he/she might administer.”* *“However, the doctor must explain the purpose of the therapy he/she adopts so that I can make a distinction too.”*	

The final area of illness-related work highlighted by this review was communication. Patients wanted to be able to communicate with their HCPs [18, 27, 34, 40] but often felt unsure of when or who to direct questions to [24, 26, 35, 36, 38]. Having a single HCP who they could talk to about all aspects of treatment was a high priority [19, 21–23, 28, 43]. Less important was the need to talk to a professional counsellor [25, 27, 36, 43].

Although a general need for information was consistent across all included studies, not all patients wanted a high volume of information. A significant minority of patients only wanted to know essential information or did not want to receive bad news [29, 31, 46]. Age may play a role in this dynamic, as multiple papers reported older people wanted less information [14, 15, 20, 22, 25, 26, 31, 44], while only a couple found no relationship [28, 34]. Timing could also be a factor, as some patients felt the amount of information received when diagnosis was overwhelming and preferred receiving information as it became relevant [34, 37, 39, 41].

### Everyday Life Work

This area of need encapsulates “the daily round of tasks that helps keep a household going”, which includes the practical tasks involved in managing an illness, along with trying to maintain the structure of life pre-diagnosis [13]. The most frequently reported social needs were about patients’ concern for their family [17, 20, 21, 26, 39, 46–48]. The importance of maintaining relationships with their partner, children or friends were all mentioned [15, 29, 37, 42, 45, 47], although notably not among patients with incurable cancer [27]. There was no consensus on whether patients wanted to discuss their cancer with loved ones; some papers found this to be highly important, others did not [20, 31, 36, 42, 49]. While there were no clear demographic or medical factors connected to this variation, Kent (2013) reported that patients whose existing relationships had been heavily affected by their diagnosis were more likely to want to talk about cancer [49].

Patients wanted to live a life they consider “normal”, reflected by the importance placed on daily living needs. The most common difficulties patients faced were coping with a lack of energy [17, 19, 21, 27, 28, 30, 36, 38, 43] and wanting to do the things they used to do [19, 21, 26, 28, 31, 39] (Box 3). Patients placed a high value on socialising and leisure time [15, 26, 32, 45] and reported a fear of being isolated or abandoned [16, 18, 20]. The importance of maintaining a job was influenced by age, with younger patients being more interested in how cancer will affect their career and their employment rights [15, 18, 20, 26, 29, 33, 39, 42].

**Table:** 

**3 - On living a “normal” life** Heidari, 2016. - *“I am eager to know when I can start my regular activities. When can I start cooking?”* **On employment** “*Returning to work is a kind of social support for me.”*	

The final practical need identified was financial, though the level of need was highly dependent on location. Patient populations with greater access to healthcare placed lower importance on financial needs [25, 27, 29, 30, 33] (Box 4). The needs in these groups related to wanting financial stability and informational support [45, 50], with low levels of interest in economic aid [34]. Patients in countries with more limited access to healthcare reported higher levels of financial stress and reliance on family for monetary support [32, 46]. This was true in all US-based studies, apart from one in which the mean income of participants was high [18, 20, 24, 28]. For these populations, financial concerns included managing bills [18, 24], bankruptcy assistance [18], paying for care [20, 32, 46] and homelessness [46]. A few financial needs were common across healthcare systems, being able to maintain a basic standard of living [27, 30, 45, 46] and helping understanding financial systems and resources [18, 25, 26, 34], though again the level of importance varied.

**Table:** 

**4 - On financial security** Hsiao, 2011. Patients with advanced cancer- *“How lucky we are to have government health insurance, otherwise the long-term hospital expenses would be a burden on my offspring.”*	

### Biographical Work

Biographical work is defined as “the work involved in defining and maintaining an identity” [13]. This involves coming to terms with and contextualising a diagnosis within a persons’ identity [42, 45]. Patients wanted to be treated as individuals [16, 19, 21, 22, 34], be reassured [19, 34], have their feelings acknowledged [19, 22], be respected [34, 45] and have their dignity preserved [47] (Box 5).

**Table:** 

**5 - On being treated like a person** Beaver, 2010. Colorectal cancer patients- *“I feel as if the doctors come and they examine you like, well you’re just a number and they have a look at you and that’s it.”* Tamburini, 2003- *“Then they moved on, without saying hello, without making eye contact.”*	

Biographical work includes dealing with the emotional impact of cancer. Feelings of despair or depression were common [19, 21, 23, 28, 30, 42, 51, 52], as well as distress and anxiety [16, 21, 28, 30, 35, 38, 43]. Patients also reported a range of fears including cancer itself [17, 21, 23, 31, 43, 51], their treatment [35, 37], dying [17, 19, 42, 52] and pain [27]. Physical changes also negatively affected patients’ sense of self [26, 27, 29, 30, 38, 42, 45–47]. Consequently, the need for relaxation and stress management was high [23, 24, 33, 48, 52].

Patients struggled to deal with the uncertainty [15, 17, 19, 28, 30, 42, 43, 46, 51] and expressed a desire for more control [17, 19–21, 27, 28, 30, 42, 43, 53]. To cope, patients placed a lot of importance on receiving support from loved ones [14, 18, 32, 35, 36, 42, 51]. However, this directly conflicted with their fear of being a burden and a perceived pressure to “stay strong” [20, 27, 37, 45, 46, 51, 53] (Box 6). Other patients were identified as a source of support for some [18, 34, 37, 49, 52], but others, especially those who were receiving follow-up or palliative care, were less interested in talking to other patients [25, 35, 45, 47]. This aligns with a reported need among terminal cancer patients to discuss things other than illness [54].

**Table:** 

**6 - On not wanting to be a burden** Beaver, 2016. Breast cancer patients- *“And then my son and he was only seventeen at that time…. And I just thought, I shouldn't have put him through this.”* **On being vulnerable** Beaver, 2016. Breast cancer patients- *“Don't like being trouble to anybody. And I think somebody should have sat me down and said “look, I think you need some help.”* Shih, 2009. - *“I was embarrassed to ask for my children’s comfort and companionship because I used to be their leader and protector.”*	

Sexuality is another part of identity that can be impacted by cancer. Patients wanted to know how cancer would impact their sex drive, sexuality [14, 17, 20, 26, 29] and their intimate relationships [14, 17, 30, 31] but often felt uncomfortable discussing these needs with their HCPs [14, 18, 20, 26] (Box 7). When ranked alongside other needs, sexuality was reported to be of lesser importance to most patients [17, 19, 21, 22, 35, 42, 43], apart from prostate cancer patients, who reported the impact on their sex drive and sexual activity as some of the most significant changes they faced [14, 30, 38] (Box 7). Higher sexuality-related needs were also identified in patients with colorectal and breast cancer, although not at the same level [22, 30]. Of the papers that looked, five out of the six studies found a relationship between age and importance of sexual identity, with younger patients having a greater need for information on sex [17, 22, 26, 31] and individuals over 40 wanting more guidance on fertility [18]. One study involving younger patients did report limited interest in sexuality; however, the majority of patients were under 18 and therefore were less likely to be sexually active [42].

**Table:** 

**7 - On difficulty asking questions about sex** Heidari, 2016- *“It is difficult to ask sexual questions, it is very hard, I am ashamed”* *“I do apologize, the question I always wanted to ask but I never did was sexual disinclination. I could never ask about this”* **On the changes in their sexuality** Grimsbo, 2011. Prostate cancer patients- *“The only thing that doesn’t function is my sex life, it’s completely dead”* *“I would have paid a lot to get my sex drive back.”*	

Much like with sexual identity, patients’ spiritual needs were not highly important when ranked alongside other domains [23, 24, 27, 28, 33, 34, 40, 45], but papers that focused solely on spirituality reported widespread need [47, 48, 52, 54–58]. There was no consensus on the importance of accessing religious resources, some papers reported a strong need for religious support [23, 32, 45, 55, 56, 59], but more papers reported low levels of interest [24, 28, 33, 48, 51, 52, 54, 57, 60]. In line with this, the most commonly reported spiritual needs were not explicitly religious. This included maintaining a sense of calm [45–48, 52, 53, 55, 56, 58, 60], staying positive or hopeful [23, 24, 32, 45, 47, 48, 57–59] and being able to appreciate or find meaning in life [32, 45, 47, 48, 55–57, 59, 60]. Generally, there was little reported interest in discussing death or dying [23, 24, 27, 42, 45, 48, 52, 60] or making sense of *why* this happened [34, 55–57]. Much like the importance of family relationships in everyday work, being with loved ones was important for patients’ spiritual wellbeing [47, 51, 53–58, 60]. However, some patients reported that being part of a religious community gave them similar support [46, 51, 53, 55, 60].

The most commonly reported religious need for patients was to pray or be prayed for [32, 46, 48, 55–57, 59]. The fact that prayer was also important for non-religious participants suggests that it may be seen as a spiritual practice for some patients. A small number of papers reported that having a relationship with God was important to patients [15, 48, 56, 57, 59], with some patients viewing God as a saviour from illness [40, 46, 59], while others felt that God caused their illness as punishment or as a test of faith [46, 47, 51] (Box 8).

**Table:** 

**Box 8: On relationship with God** Hsiao, 2011. “*My disease resulted from previous bad karma”* Elsner, 2012. *“I believe that God will cure my disease, he is the one who is protecting me from worsening of the disease.”*	

Cultural factors may also influence spiritual needs. The afterlife was found to be an important concern for some patients [52, 53, 55], but not if their culture had little belief in the concept [47]. In the same way, having a legacy was a key need in one paper due to the importance of continuity after death in that culture [53].

## Discussion

This is the first review to synthesise data about cancer patients’ supportive needs across all populations and cancer types. There was remarkable consistency in the needs identified, and these were well explained by the Corbin & Strauss model of managing a chronic condition [13]. Almost all studies confirmed patients’ need for high-quality, comprehensible and timely information about their illness, treatments and how best to manage their symptoms. Such information was necessary for patients to undertake illness-related, everyday living and biographical work. In addition, patients needed support in dealing with emotional issues, including existential uncertainty, changing relationships with friends and family and practical support with everyday tasks.

### Previous Literature

This review confirms the findings of previous reviews focused on specific types of need or specific populations. The most common needs identified as illness-related work in this study correspond to key informational needs highlighted in previous reviews [61, 62]. The spiritual needs discussed have also been found to be key in improving psycho-spiritual wellbeing in other research [63]. While our review did not assess the ability of current care models to meet these needs, it is noteworthy that the key needs we identified have been found to frequently go unmet in other research [64, 65].

### Strengths and Limitations

The main strength of this study is its inclusive nature, looking across all populations and all types of cancer. This, combined with the theoretical underpinning and use of the Corbin & Strauss model, provides reassurance about the overall transferability of these findings to other clinical populations.

The main limitation pertains to the scope of the primary literature, with most of the studies coming from high-income countries, and only 7 papers from low- or middle-income countries. While the nature of patients’ financial needs were clearly dependent on country, setting may also influence other needs in less direct ways, limiting how universal the findings are. Additionally, the majority of studies used opportunistic sampling so may not accurately capture the needs of the general cancer population. Most included studies were not longitudinal and therefore could not analyse how patients’ concerns changed over time. Finally, potentially relevant demographic information was not always collected. For example, only one of the papers that examined sexuality collected information about sexual orientation, and only a couple of studies that measured financial need recorded socioeconomic status.

## Conclusions

This review highlights a number of underlying issues that affect cancer patients. These findings are consistent with the previous literature and fit well with multiple chronic illness frameworks, which suggests that they are robust enough to inform best practice. The most common needs identified support the argument for empowering people with cancer through a patient-centred form of care.

Priorities for practice should be to ensure patients understand their illness and what they can expect throughout their treatment pathway. Supportive care should work to enable patients to live a life they recognise as “normal” and help them maintain their closest relationships. HCPs should ensure that patients always feel that they are being treated as individuals and know who to go to when they have questions. These key needs should be addressed as a first step to provide a strong basis of care before providing more individualised support.

Further research should focus on how to ensure these needs are addressed effectively. Evaluation of supportive care interventions should remain focused on the experiences of patients to allow them to have a voice in their care. Additional research on when different needs arise over the disease progression would help ensure that resources are provided only when needed.

## Supplementary Information

ESM 1 (DOCX 58 kb)

ESM 2 (DOCX 118 kb)
