# Supplementary material for: The Supportive Care Needs of Cancer Patients: a Systematic Review
Source: J Cancer Educ. 2021 Jan 25;36(5):899–908. doi: 10.1007/s13187-020-01941-9 (PMC8523012; doi:10.1007/s13187-020-01941-9)
Supplement: Supplementary file 1 — (DOCX 58 kb) [file 13187_2020_1941_MOESM1_ESM.docx]

**Appendix 1- Search strategy for the 3 online databases searched**

1. Information* need*.mp. [mp=title, abstract, heading word, drug trade name, original title, device manufacturer, drug manufacturer, device trade name, keyword, floating subheading word, candidate term word]

2. Emotion* stress*.mp. [mp=title, abstract, heading word, drug trade name, original title, device manufacturer, drug manufacturer, device trade name, keyword, floating subheading word, candidate term word]

3. Emotion* need*.mp. [mp=title, abstract, heading word, drug trade name, original title, device manufacturer, drug manufacturer, device trade name, keyword, floating subheading word, candidate term word]

4. Spiritual need*.mp. [mp=title, abstract, heading word, drug trade name, original title, device manufacturer, drug manufacturer, device trade name, keyword, floating subheading word, candidate term word]

5. Social need*.mp. [mp=title, abstract, heading word, drug trade name, original title, device manufacturer, drug manufacturer, device trade name, keyword, floating subheading word, candidate term word]

6. 1 or 2 or 3 or 4 or 5

7. Neoplasm/

8. 6 and 7
