# Supplementary material for: The Supportive Care Needs of Cancer Patients: a Systematic Review
Source: J Cancer Educ. 2021 Jan 25;36(5):899–908. doi: 10.1007/s13187-020-01941-9 (PMC8523012; doi:10.1007/s13187-020-01941-9)
Supplement: Supplementary file 2 — (DOCX 118 kb) [file 13187_2020_1941_MOESM2_ESM.docx]

| Author | | Title | Year of Publication | Journal of publication | Location of study | Population studied |
| --- | --- | --- | --- | --- | --- | --- |
| Astrow, A. B. | Is failure to meet spiritual needs associated with cancer patient's perceptions of quality of care and their satisfaction with care? | | 2007 | Journal of Clinical Oncology | USA | 369 outpatients of a cancer centre |
| Beaver, K. | An exploratory study of the follow-up care needs of patients treated for colorectal cancer | | 2010 | Journal of Clinical Nursing | UK | 27 colorectal cancer patients at a specialist outpatient clinic |
| Beaver, K. | Exploring patient experiences of neo-adjuvant chemotherapy for breast cancer | | 2016 | European Journal of Oncology Nursing | UK | 20 women who had completed neo-adjuvant chemo for breast cancer |
| Beesley, V. | A tsunami of unmet needs: Pancreatic and ampullary cancer patient's supportive care needs and use of community and allied health services | | 2015 | Pyscho-Oncology | Australia | 136 patients with ampullary or pancreatic cancer |
| Boberg, E. | Assessing the unmet information, support and care delivery needs of men with prostate cancer | | 2003 | Patient Education and Counselling | USA | 204 patients diagnosed with prostate cancer over 4 states |
| Buzgova, R. | Association between unmet needs and quality of life in hospitalised cancer patients no longer receiving anti-cancer treatment | | 2013 | European Journal of Cancer Care | Czech Republic | 93 patients from the oncology department of University Hospital in Ostrava for whom cancer therapy was terminated because it was no longer effective |
| Choi, K. H. | Cancer patients' informational needs on health promotion and related factors: a multi-institutional, cross-sectional study in Korea | | 2010 | Supportive Care in Cancer | Korea | 2661 patients treated for cancer at 10 Korean National Cancer Centre over 2 months |
| Decker, C. | Information needs of adolescents with cancer | | 2004 | Journal of Paediatric Oncology Nursing | USA | 39 adolescents 1-3 years post-diagnosis and 74 newly diagnosed |
| Dedeli, O. | Assessing the spiritual needs and practices of oncology patients in Turkey | | 2015 | Holistic Nursing Practice | Turkey | 230 patients from state hospital |
| Derdiarian, A. K. | Informational needs of recently diagnosed cancer patients | | 1986 | Nursing Research | USA | 60 recently diagnosed cancer patients, not late stage |
| Doorenbos, A. Z. | Satisfaction with telehealth for cancer support groups in rural American Indian and Alaska Native communities | | 2010 | Clinical Journal of Oncology Nursing | USA | 50 cancer survivors who attended support group meetings and were either American Indian or native Alaskan |
| Dubey, C. | Resilience and unmet supportive care needs in patients with cancer during early treatment: A descriptive study | | 2015 | European Journal of Oncology Nursing | Switzerland | 68 cancer patients 4-15 weeks post-diagnosis of a solid tumour cancer |
| Elsner, F. | Psychosocial and spiritual problems of terminally ill patients in Kerala, India | | 2012 | Future Oncology | India | 37 cancer patients receiving palliative care |
| Ghahramanian, A. | Spiritual needs of patients with cancer referred to Alinasab and Shahid Ghazi Tabatabaie Hospitals of Tabriz, Iran | | 2016 | Pyscho-Oncology | Iran | 200 cancer patients across 2 hospitals diagnosed with cancer |
| Giacalone, A. | What elderly cancer patients want to know? Differences among elderly and young patients | | 2007 | Pyscho-Oncology | Italy | 122 cancer patients over 65 years old and 52 cancer patients between the ages of 18-40 from a single hospital institute |
| Goldfarb, M. | Unmet information and support needs in newly diagnosed thyroid cancer: Comparison of adolescent/young adults (AYA) and older patients | | 2014 | Journal of Cancer Survivorship | USA | 1059 patients from on an online thyroid cancer survival group |
| Grimsbo, G. H. | Left hanging in the air: Experiences of living with cancer as expressed through e-mail communications with oncology nurses | | 2011 | Cancer Nursing | Norway | 276 messages from 60 breast and prostate cancer patients |
| Hampton, D. M. | Spiritual needs of persons with advanced cancer | | 2007 | American Journal of Hospice and Palliative Medicine | USA | 90 patients with advanced cancer admitted to two different hospices in South Florida |
| Hasegawa, T. | Prevalence of unmet needs and correlated factors in advanced-stage cancer patients receiving rehabilitation | | 2016 | Supportive Care in Cancer | Japan | 45 patients using cancer rehabilitation services |
| Hawkins, N. A. | Informational needs of patients and perceived adequacy of information available before and after treatment of cancer | | 2008 | Journal of Psychosocial Oncology | USA | 731 recently diagnosed patients with life expectancy of longer that 10 months |
| Heidari, H. | Cancer patients' informational needs: Qualitative content analysis | | 2015 | Journal of Cancer Education | Iran | 25 in-hospital patients in two locations |
| Hocker, A. | Exploring spiritual needs and their associated factors in an urban sample of early and advanced cancer patients | | 2014 | European Journal of Cancer Care | Germany | 285 outpatients with multiple cancer types at various stages |
| Hsiao, S. M. | An exploration of spiritual needs of Taiwanese patients with advanced cancer during the therapeutic process | | 2010 | Journal of Clinical Nursing | Taiwan | 33 patients with advanced cancer from a medical centre and a teaching hospital |
| James-Marin, G. | Information needs of cancer patients and survivors regarding diet, exercise and weight management: A qualitative study | | 2014 | European Journal of Cancer Care | Australia | 6 patients and 1 cancer survivor of any diagnosis |
| Kent, E. E. | Talking about cancer and meeting peer survivors: Social information needs of adolescents and young adults | | 2013 | Journal of Adolescent and Young Adult Oncology | USA | 525 AYA cancer patients 6-14 months post-diagnosis aged 15-39 |
| Korner, A. | Supportive care needs and distress in patients with non-melanoma skin cancer: Nothing to worry about? | | 2016 | European Journal of Oncology Nursing | Canada | 60 patients with squamous or basal cell carcinoma |
| Li, W. W. | Interpreting differences in patterns of supportive care needs between breast cancer and patients with colorectal cancer | | 2013 | Pyscho-Oncology | China | 210 out patients with colorectal and breast cancer |
| Masika, G. M. | Health-related quality of life and needs of care and support of adult Tanzanians with cancer: A mixed-methods study | | 2012 | Health and Quality of Life Outcome | Tanzania | 101 patients admitted to a cancer institute |
| Moadel, A. B. | Psychosocial needs assessment among an underserved, ethnically diverse cancer patient population | | 2006 | Cancer | USA | 248 outpatients in New York |
| Moody, K. | Psychosocial needs of ethnic minority, inner-city, paediatric cancer patients | | 2011 | Supportive Care in Cancer | USA | 112 families with children who have any form of cancer |
| Murray, S. A. | Exploring the spiritual needs of people dying of lung cancer or heart failure: A prospective qualitative interview study of patients and their carers | | 2004 | Palliative Medicine | UK | 20 patients with inoperable lung cancer |
| Neumann, M. | Identifying and predicting subgroups of information needs among cancer patients: an initial study using latent class analysis | | 2010 | Supportive Care in Cancer | Germany | 326 cancer patients of 6 types- bronchial, oesophageal, colorectal, breast, prostate and skin cancer. |
| Ng, R. | Unmet psychosocial needs among cancer patients undergoing ambulatory care in Singapore | | 2011 | Supportive Care in Cancer | Singapore | 535 cancer patients of 3 types- breast, gynaecological and colorectal. |
| O'Connor, G. | Exploring the information needs of patients with cancer of the rectum | | 2010 | European Journal of Oncology Nursing | UK | 40 patients who have been treated for or undergone surgery for rectal cancer |
| Rainbird, K. | The needs of patients with advanced, incurable cancer | | 2009 | British Journal of Cancer | Australia | 246 with advanced terminal cancer but who are not receiving palliative care (LE. 3 months- 2 years) |
| Sanders, S. L. | Supportive care needs in patients with lung cancer | | 2010 | Pyscho-Oncology | USA | 109 patients from two medical centres with primary non-small cell or small cell lung cancer. |
| Sharma, R. K. | The Spiritual Needs Assessment for Patients (SNAP): Development and validation of a comprehensive instrument to assess unmet spiritual needs | | 2012 | Journal of Pain and Symptom Management | USA | 47 cancer patients of any diagnosis |
| Shihi, F. J. | Spiritual needs of Taiwan’s' older patients with terminal cancer | | 2009 | Oncology nursing forum | Taiwan | 35 cancer patients over 65 with terminal cancer and a L.E of less than 3 months |
| Tamburini, M. | Cancer patients' needs during hospitalisation: a quantitative and qualitative study. | | 2003 | BioMed Central Cancer | Italy | 182 cancer patients that had been hospitalised less than 48 hours (excluding paediatric patients) |
| Taylor, E. J. | Spiritual needs of patients with cancer and family caregivers | | 2003 | Cancer Nursing | USA | 21 both in and out cancer patients of either Euro-American or African-American background. |
| Taylor, E. J. | Prevalence and associated factors of spiritual needs among patients with cancer and family caregiver | | 2006 | Oncology nursing forum | USA | 156 both in and out cancer patients of either Euro-American or African-American background. |
| Van Weert, J. C. | Older cancer patients' information and communication needs: What they want is what they get? | | 2013 | Patient Education and Counselling | Netherlands | 155 cancer patients over 65 receiving chemotherapy for the first time. |
| Vilalta, A. | Evaluation of spiritual needs of patients with advanced cancer in a palliative care unit | | 2014 | Journal of Palliative Medicine | Spain | 50 patients diagnosed with advanced or end-stage cancers being monitored by an outpatient clinic |
| Whelan, T. J. | The supportive care needs of newly diagnosed cancer patients attending a regional cancer centre | | 1997 | Cancer | Canada | 134 patients diagnosed with lung, breast, prostate, colorectal, head and neck and non- melanoma skin cancer |
| White, K. | Mapping the psychosocial and practical support needs of cancer patients in Western Australia | | 2012 | European Journal of Cancer Care | Australia | 786 cancer patients across Western Australia on the national registry diagnosed 6 months to 2 years ago |
| Wong, R. K. | What do patients living with advanced cancer and their carers want to know? - A needs assessment | | 2002 | Supportive Care in Cancer | Canada | 71 patients receiving palliative radiotherapy |
